# An analysis of ranibizumab treatment and visual outcomes in real-world settings: the UNCOVER study

**DOI:** 10.1007/s00417-017-3890-8

**Published:** 2018-03-03

**Authors:** Bora Eldem, Timothy Y. Y. Lai, Nor Fariza Ngah, Brendan Vote, Hyeong Gon Yu, Alban Fabre, Arthur Backer, Nathan J. Clunas

**Affiliations:** 1 Ophthalmology Department, Hacettepe University School of Medicine, 06100 Ankara, Samanpazarı Turkey; 2 Department of Ophthalmology and Visual Sciences, The Chinese University of Hong Kong, Hong Kong, China; 3 Department of Ophthalmology, Hospital Selayang, Selayang Selangor, Malaysia; 4 Launceston Eye Institute, South Launceston, Australia; 5 Department of Ophthalmology, Seoul National University College of Medicine, Seoul, South Korea; 6 IQVIA, Barcelona, Spain; 7 Novartis Pharma AG, Basel, Switzerland

**Keywords:** Observational study, Ranibizumab, Visual acuity, Tomography, Optical coherence

## Abstract

**Purpose:**

To describe intravitreal ranibizumab treatment frequency, clinical monitoring, and visual outcomes (including mean central retinal thickness [CRT] and visual acuity [VA] changes from baseline) in neovascular age-related macular degeneration (nAMD) in real-world settings across three ranibizumab reimbursement scenarios in the Middle East, North Africa, and the Asia–Pacific region.

**Methods:**

Non-interventional multicenter historical cohort study of intravitreal ranibizumab use for nAMD in routine clinical practice between April 2010 and April 2013. Eligible patients were diagnosed with nAMD, received at least one intravitreal ranibizumab injection during the study period, and had been observed for a minimum of 1 year (up to 3 years). Reimbursement scenarios were defined as self-paid, partially-reimbursed, and fully-reimbursed.

**Results:**

More than three-fourths (*n* = 2521) of the analysis population was partially-reimbursed for ranibizumab, while 16.4% (*n* = 532) was fully-reimbursed, and 5.8% was self-paid (*n* = 188). The average annual ranibizumab injection frequency was 4.1 injections in the partially-reimbursed, 4.7 in the fully-reimbursed and 2.6 in the self-paid populations. The average clinical monitoring frequency was estimated to be 6.7 visits/year, with similar frequencies observed across reimbursement categories. On average, patients experienced VA reduction of −0.7 letters and a decrease in CRT of −44.4 μm. The greatest mean CRT change was observed in the self-paid group, with −92.6 μm.

**Conclusions:**

UNCOVER included a large, heterogeneous ranibizumab-treated nAMD population in real-world settings. Patients in all reimbursement scenarios attained vision stability on average, indicating control of disease activity.

**Electronic supplementary material:**

The online version of this article (10.1007/s00417-017-3890-8) contains supplementary material, which is available to authorized users.

## Introduction

Age-related macular degeneration (AMD) causes progressive, severe, irreversible vision loss and is the leading cause of blindness in individuals older than 50 years [1]. The neovascular form of AMD (nAMD) is characterized by choroidal neovascularization. Although nAMD is much less common than other forms of AMD, 80% to 90% of severe vision loss related to AMD is attributable to this form [2, 3].

Pooled prevalence estimates from a meta-analysis of Asian populations aged 40 to 79 years in Singapore, Japan, China, Taiwan, Brazil, the United States, and India were 6.8% for early AMD and 0.56% for late AMD [4]. Projections by Wong and colleagues suggested that in the year 2040, there will be 113 million cases of any form of AMD in Asia, and 39 million cases each in Africa, Latin America, and the Caribbean [5].

Recent literature has also revealed specific subtypes of nAMD such as polypoidal choroidal vasculopathy (PCV) [6] and retinal angiomatous proliferation (RAP). PCV prevalence among patients with nAMD is estimated to be 22% to 55% in Asian populations and 4% to 14% in Caucasian patients, while the RAP subtype may be present in up to 15% of all newly diagnosed nAMD cases [7].

Ranibizumab (Lucentis, Novartis Pharma AG, Basel, Switzerland; Genentech Inc., San Francisco, CA, USA), an anti-vascular endothelial growth factor (anti-VEGF) agent specifically designed for ocular use, is licensed for the treatment of nAMD worldwide. The efficacy and safety of monthly intravitreal injections of ranibizumab have been demonstrated in several clinical trials, notably MARINA and ANCHOR [8, 9]. LUMINOUS, a non-interventional study on safety and effectiveness of ranibizumab, was conducted in Asia, Australia, Europe, and North and South America, and provides further support for its positive safety profile. A retrospective, pooled safety analysis of data from 4 European nAMD registries in the LUMINOUS program exhibited a favorable 1-year safety profile for ranibizumab in routine clinical practice [10].

The recommended regimen of monthly administration schedules of ranibizumab that were used in early clinical trials has proved burdensome to patients and the healthcare system due to factors such as cost of treatment. Subsequent investigations have examined the efficacy of alternative ranibizumab treatment regimens with fewer injections, such as “pro re nata” (PRN) regimen, in which the drug is administered on an as-needed basis, and “treat and extend” (T&E) regimen, in which the drug is administered on a regular basis until the condition stabilizes; after stabilization, the interval between additional treatments is based on changes in specific clinical parameters [11–14]. The HARBOR clinical trial, wherein patients received 3-month loading doses of ranibizumab followed by PRN regimen, demonstrated clinically meaningful visual improvement at 12 and 24 months from baseline [15, 16].

While some publications have demonstrated the efficacy, positive safety profile, and cost-effectiveness associated notably with PRN treatment, to the best of our knowledge, no study has yet examined ranibizumab use and visual outcomes by treatment reimbursement scenarios. The non-interventional UNCOVER (UNraveling nAMD real life Clinical management and Outcome with intraVitrEal Ranibizumab injection) historical cohort study was designed to describe ranibizumab treatment frequency, visual outcomes, and their correlation, in addition to clinical monitoring frequency of patients treated for nAMD in real-world settings in the Middle East, North Africa, and the Asia-Pacific region. Patients included in UNCOVER were categorized by ranibizumab reimbursement scenario (fully-reimbursed, partially-reimbursed, or self-paid).

## Material and methods

UNCOVER was a multicenter historical cohort study of intravitreal ranibizumab use for nAMD in routine clinical practice based on the abstraction of medical records. Among the 60 ophthalmology centers that participated in this study, three were government hospitals, three were primary care settings, ten were private institutions, and 44 were teaching or university hospitals. Data for nAMD patients were extracted from their medical records in the following ten countries: Algeria, Australia, Hong Kong, India, Malaysia, Saudi Arabia, Singapore, South Korea, Thailand, and Turkey.

The objectives of this study were to describe ranibizumab treatment frequency, pattern of care (i.e., clinical monitoring frequency), and clinical outcomes (including mean central retinal thickness [CRT] and visual acuity [VA] changes from baseline) across three reimbursement scenarios. Those scenarios were defined as *fully-reimbursed* when the cost of ranibizumab was paid in full by insurance (private, government, etc.), *partially-reimbursed* when it was paid partially by insurance and the patient, and *self-paid* when it was paid entirely by the patient. The correlation between the frequency of ranibizumab injections and VA or CRT was also explored.

The study protocol was approved by ethics committees or institutional review boards in each participating country, and by the Ministry of Health in Algeria. Informed consent was obtained from individual participants included in the study where required. A waiver of informed consent was obtained for all sites in Algeria, Malaysia, Singapore, Saudi Arabia and Turkey, and a subset of sites in Hong Kong, India, South Korea, and Thailand.

### Study participants

All patients with a diagnosis of nAMD who received at least one intravitreal ranibizumab injection at any time between April 2010 and April 2013 and had been observed for a minimum of 1 year (up to 3 years) were eligible for this study. The observation period for each patient started with the first ranibizumab injection recorded during the study period (baseline) and ended at either last monitoring of ranibizumab treatment or when the patient switched to another anti-VEGF treatment. Patients were excluded from participation if they received intravitreal bevacizumab or other anti-angiogenic agents intermittently or concomitantly during the study period, or if they received treatment with a ranibizumab dose other than 0.5 mg.

### Study outcomes

Patients were characterized by ranibizumab reimbursement scenario, medical history, date of nAMD diagnosis and prior nAMD treatment. Concomitant nAMD therapies (other than additional anti-VEGF therapy), adverse events (AEs) as reported in the medical records, and changes in nAMD therapy (including names of therapies and reason for switching) were also collected at the end of the observation period. Dates of administration of 0.5 mg intravitreal injections of ranibizumab (by eye) were collected to assess the frequency of ranibizumab treatment (injections per year), while measurements of VA (in letters) and optical coherence tomography measures of CRT (in μm) were collected to assess the clinical monitoring frequency and the changes in VA and CRT from baseline. Clinical monitoring frequency was derived from the number of VA and CRT assessments per year.

### Statistical analysis

The final analysis population included all eligible patients. All analyses were stratified by reimbursement scenario. All statistical tests were for exploratory purposes and were 2-sided (alpha < 0.05). Continuous variables were described by mean and standard deviation (±). A patient-level analysis (the patient as the analysis unit) was conducted on various characteristics such as age, sex, race, ethnicity, country, relevant medical history/comorbidities, and AEs. An eye-level analysis (the eye as the analysis unit) was also conducted to assess: duration from nAMD diagnosis to first ranibizumab injection reported in the study, nAMD history, ranibizumab treatment and clinical monitoring frequencies, number of VA and CRT assessments, VA and CRT change from baseline. Baseline assessments for VA and CRT were defined as measurements occurring on the day of the first recorded ranibizumab injection, or at any time prior to the first recorded injection during the observation period. The association between changes in VA and CRT from baseline to the last assessment and frequency of ranibizumab injections was examined using the Spearman rank correlation coefficient among the eye-population. A best subsets regression model using R-squared methods was conducted to identify variables associated with changes in VA and CRT from baseline to the last assessment. The eyes of an individual were considered independent given the pathophysiology of nAMD.

Two subgroups were identified for the analyses. Subgroup A: patients with the last recorded ranibizumab injection occurring between 13 and 24 months after the first recorded injection; and subgroup B: patients with the last recorded injection occurring between 25 and 36 months after the first recorded injection. All analyses were performed with the statistical software SAS® (SAS Institute, Cary, NC, USA).

## Results

A total of 3445 eligible patients were enrolled. Among those, 3241 patients meeting all inclusion criteria were included in the final analysis population, corresponding to 3725 treated eyes (overall eye-population).

### Baseline characteristics

Approximately three-quarters of the analysis population (77.8%, *n* = 2521) were partially-reimbursed for ranibizumab, 16.4% were (*n* = 532) fully-reimbursed, and 5.8% self-paid (*n* = 188) (Table 1). The majority of patients were from Turkey (40.1%), followed by Australia (27.5%), and South Korea (20.4%). Turkish patients made up the highest proportion (51.1%) of the partially-reimbursed group, Australian patients made up the largest proportion (40.0%) of the fully-reimbursed group, and Hong Kong patients made up 61.2% of the self-paid group (Table 1).

**Table 1:** Analysis population by country

Analysis population, no. (%)	Ranibizumab medical coverage			All combined (*n* = 3241)
	Fully-reimbursed (*n* = 532)	Partially-reimbursed (*n* = 2521)	Self-paid (*n* = 188)	
Algeria	38 (7.1%)	0	0	38 (1.2%)
Australia	213 (40.0%)	671 (26.6%)	7 (3.7%)	891 (27.5%)
Hong Kong	31 (5.8%)	12 (0.5%)	115 (61.2%)	158 (4.9%)
India	9 (1.7%)	4 (0.2%)	26 (13.8%)	39 (1.2%)
Republic of Korea	113 (21.2%)	538 (21.3%)	9 (4.8%)	660 (20.4%)
Malaysia	63 (11.8%)	1 (0.0%)	22 (11.7%)	86 (2.7%)
Saudi Arabia	12 (2.3%)	0	0	12 (0.4%)
Singapore	0	6 (0.2%)	1 (0.5%)	7 (0.2%)
Thailand	48 (9.0%)	0	2 (1.1%)	50 (1.5%)
Turkey	5 (0.9%)	1289 (51.1%)	6 (3.2%)	1300 (40.1%)

The mean observation period was 22.3 (± 7.83) months in the overall population, and was similar across reimbursement categories (Table 2). Gender was similarly distributed among the fully- and partially-reimbursed groups (53.2% and 50.3% female respectively), while males comprised the majority of the self-paid group (65.4% male) (Table 1). Similar age distributions were observed across reimbursement groups, with a mean age of 73.5 (± 9.98) years overall (Table 2). A higher proportion of the self-paid group had eye-related medical history events compared to the two other reimbursement categories, notably PCV (33% versus approximately 5%, *n* = 62 versus *n* = 138 and *n* = 27 in the partially- and fully-reimbursed populations respectively) (Table 2).

**Table 2:** Patient baseline characteristics

Patient baseline characteristics	Ranibizumab medical coverage			All combined (*n* = 3241)
	Fully-reimbursed (*n* = 532)	Partially-reimbursed (*n* = 2521)	Self-paid (*n* = 188)	
Mean observation period in months [SD]	23.9 [8.24]	22.0 [7.79]	21.9 [6.57]	22.3 [7.83]
Mean age in years [SD]	75.5 [10.10]	73.2 [9.87]	71.9 [10.38]	73.5 [9.98]
Female, no. (%)	283 (53.2%)	1267 (50.3%)	65 (34.6%)	1615 (49.8%)
Race, no. (%)				
Caucasian	230 (43.2%)	1259 (49.9%)	7 (3.7%)	1496 (46.2%)
Asian	264 (49.6%)	582 (23.1%)	175 (93.1%)	1021 (31.5%)
Other	38 (7.1%)	678 (26.9%)	6 (3.2%)	722 (22.3%)
Medical history^a^, no. (%)				
Cataract	227 (42.7%)	822 (32.6%)	98 (52.1%)	1147 (35.4%)
Pseudophakia	116 (21.8%)	557 (22.1%)	66 (35.1%)	739 (22.8%)
Polypoidal choroidal vasculopathy	27 (5.1%)	138 (5.5%)	62 (33.0%)	227 (7.0%)
Hypertension	273 (51.3%)	1093 (43.4%)	84 (44.7%)	1450 (44.7%)
Diabetes mellitus	95 (17.9%)	372 (14.8%)	41 (21.8%)	508 (15.7%)
Dyslipidemia	97 (18.2%)	244 (9.7%)	17 (9.0%)	358 (11.0%)
Other cardiovascular events	62 (11.7%)	226 (9.0%)	16 (8.5%)	304 (9.4%)

^a^Most prevalent medical conditions are presented (representing at least 10% of one or more reimbursement categories)

*SD*, standard deviation

On average, treated eyes (*n* = 3725) were diagnosed with nAMD 1 year prior to the observation period start. Half of the eyes were diagnosed with nAMD approximately 1 month prior to the observation period start, irrespective of the reimbursement category (median of 0.1 years). Six hundred and twenty-two eyes (622, 16.7%) received another nAMD treatment prior to starting ranibizumab. Photodynamic therapy (PDT) was the most common concomitant nAMD treatment in the self-paid group (16.0%) (Table 3).

**Table 3:** Eye baseline characteristics

Eye baseline characteristics	Ranibizumab medical coverage			All combined (*n* = 3725)
	Fully-reimbursed (*n* = 580)	Partially-reimbursed (*n* = 2954)	Self-paid (*n* = 200)	
Right eye, no. (%)	304 (52.4%)	1485 (50.4%)	99 (49.5%)	1888 (50.7%)
Mean time since diagnosis in years [SD]	0.8 [1.30]	1.1 [2.02]	0.8 [1.81]	1.0 [1.91]
Median time since diagnosis in years [IQR]	0.1 [0.0–1.1]	0.1 [0.0–1.4]	0.1 [0.0–0.8]	0.1 [0.0–1.3]
Treatment received prior to ranibizumab, no. (%)	130 (22.4%)	438 (14.9%)	54 (27.0%)	622 (16.7%)
Photodynamic therapy	70 (12.1%)	133 (4.5%)	32 (16.0%)	235 (6.3%)
Other anti-VEGF	65 (11.2%)	273 (9.3%)	13 (6.5%)	351 (9.4%)
Other	14 (2.4%)	72 (2.4%)	17 (8.5%)	103 (2.8%)
Surgery	1 (0.2%)	40 (1.4%)	2 (1.0%)	43 (1.2%)
Laser	11 (1.9%)	25 (0.8%)	3 (1.5%)	39 (1.0%)
Steroid	5 (0.9%)	8 (0.3%)	1 (0.5%)	14 (0.4%)
Mean baseline VA in letters [SD]	61.4 [24.18]	62.5 [26.83]	57.7 [24.99]	62.1 [26.38]
Mean baseline CRT in μm [SD]	348.7 [158.33]	317.1 [128.07]	420.1 [198.12]	327.6 [139.93]

*Anti-VEGF*, anti-vascular endothelial growth factor; *CRT*, central retinal thickness; *IQR*, interquartile range; *SD*, standard deviation; *VA*, visual acuity

### Ranibizumab use and monitoring

**Fig. 1:**
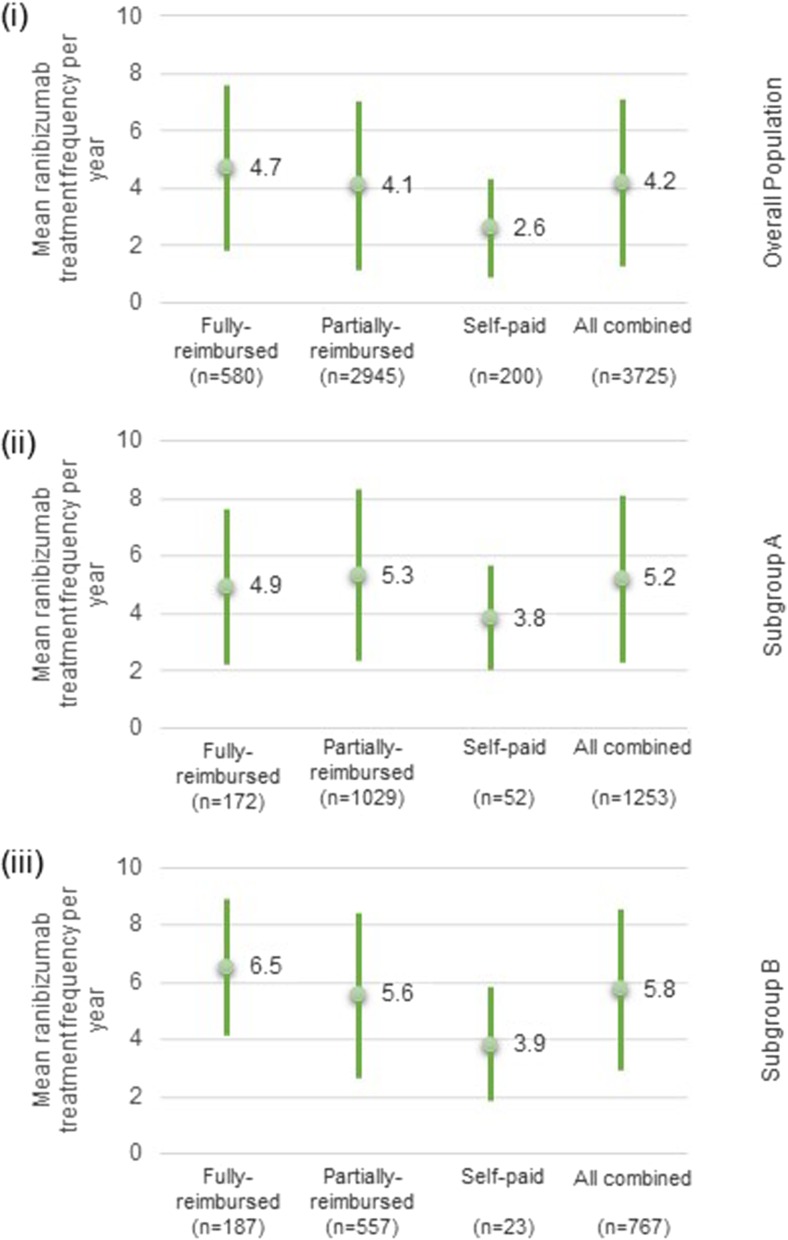
Mean ranibizumab treatment frequency per year. (i) Overall population. (ii) Subgroup A: patients with a last recorded ranibizumab injection occurring between 13 and 24 months after the first recorded ranibizumab injection. (iii) Subgroup B: patients with a last recorded ranibizumab injection occurring between 25 and 36 months after the first recorded injection

Mean yearly ranibizumab injection frequency was 2.6 (±1.71) in the self-paid category, 4.1 (±2.94) in the partially-reimbursed population (mainly driving the overall eye-population results), and 4.7 (±2.87) in the fully-reimbursed population (Fig. 1). The clinical monitoring frequency was approximately 7 visits per year on average in the overall eye-population. Similar figures were observed across the reimbursement categories (Table 4).

**Table 4:** Clinical monitoring in the overall eye-population and in the subgroups (A and B)

Clinical monitoring	Ranibizumab medical coverage			All combined
	Fully-reimbursed	Partially-reimbursed	Self-paid	
Overall eye-population, no.	580	2954	200	3725
Mean clinic visit number [SD]	6.6 [3.43]	6.7 [3.58]	6.9 [2.91]	6.7 [3.53]
Subgroup A^a^ – eye-population, No.	172	1029	52	1253
Mean clinic visit number [SD]	7.9 [3.91]	7.5 [3.84]	7.8 [2.36]	7.6 [3.81]
Subgroup B^a^ – eye-population, No.	187	557	23	767
Mean clinic visit number [SD]	5.2 [2.31]	6.4 [3.17]	7.9 [3.23]	6.1 [3.04]

^a^Subgroup A: patients with a last recorded ranibizumab injection occurring between 13 and 24 months after the first recorded ranibizumab injection. Subgroup B: patients with a last recorded ranibizumab injection occurring between 25 and 36 months after the first recorded injection

*SD,* standard deviation

### Visual outcomes

**Fig. 2:**
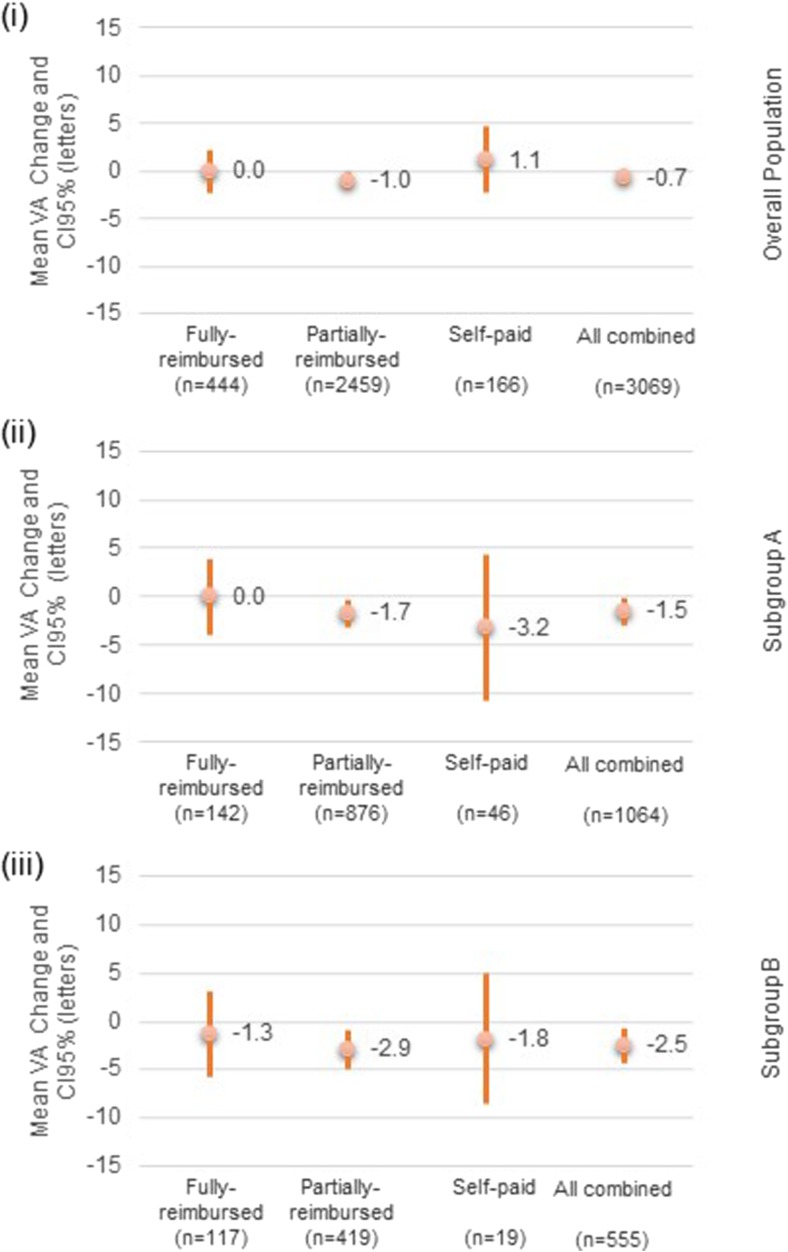
Mean visual acuity change. Note: visual acuity (VA) change was assessed on 3069 eyes in the overall population, on 1064 eyes in the subgroup A, and on 555 eyes in the subgroup B. (i) Overall population. (ii) Subgroup A: patients with a last recorded ranibizumab injection occurring between 13 and 24 months after the first recorded ranibizumab injection. (iii) Subgroup B: patients with a last recorded ranibizumab injection occurring between 25 and 36 months after the first recorded injection

**Fig. 3:**
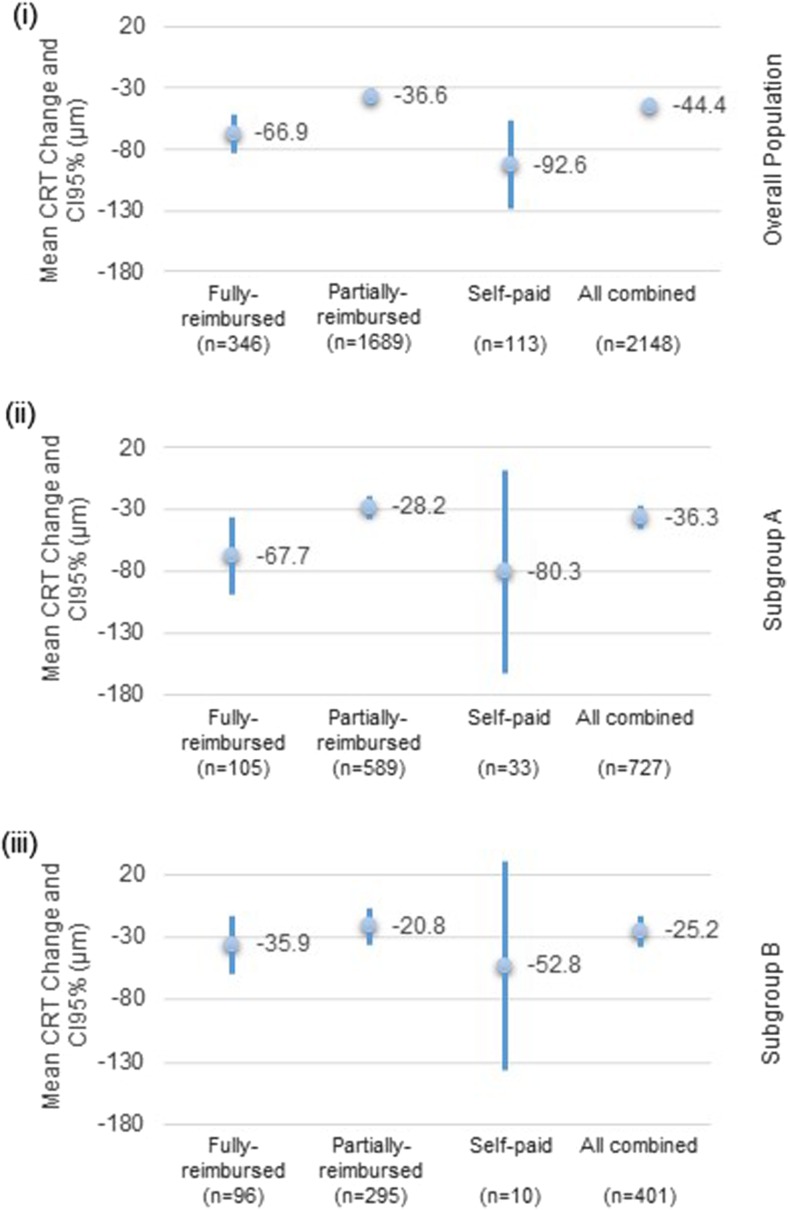
Mean central retinal thickness change. Note: Central retinal thickness (CRT) change was assessed on 2148 eyes in the overall population, 727 eyes in the subgroup A, and on 401 eyes in the subgroup B. (i) Overall population. (ii) Subgroup A: patients with a last recorded ranibizumab injection occurring between 13 and 24 months after the first recorded ranibizumab injection. (iii) Subgroup B: patients with a last recorded ranibizumab injection occurring between 25 and 36 months after the first recorded injection

Among the overall eye-population, the eyes (82.4%, *n* = 3069) with at least two VA assessments had a mean VA change of −0.7 (±22.02) letters from baseline. The mean VA change from baseline was lower than 5 letters across the different reimbursement categories (Fig. 2). For 60.4% of eyes assessed (*n* = 1853), no VA change (*n* = 624) or a gain of letters was observed (*n* = 583 gained lower than 15 letters and *n* = 646 gained 15 letters or greater); whereas 21.4% of eyes assessed lost 15 letters or more (*n* = 657). Nearly two-thirds (57.7%, *n* = 2148) of the overall eye-population had at least two CRT assessments, and a mean CRT change of −44.4 (±140.69) μm was observed from baseline. CRT decreased across all reimbursement categories, with the largest mean decrease observed in the self-paid group (−92.6 [±196.93] μm) (Fig. 3).

In the univariate analysis, there was a weak correlation between the mean VA change or CRT change and the frequency of ranibizumab injections over the observation period, with a correlation coefficient of 0.042 (*P* = 0.019) and 0.010 (*P* = 0.757) for VA and CRT change respectively.

The multivariate regression model showed that, irrespective of the patient profile and length of the observational period, the VAS increased by +0.66 letters per additional ranibizumab injection per year on average (*P* < 0.001) (Online Resource 1). Considering the different reimbursement scenarios, the CRT decreased by −22.70 μm on average in the partially-reimbursed group (*P* = 0.007), while it decreased by −40.41 μm in the fully-reimbursed group (*P* = 0.004) on average when adjusting to baseline co-variates (Online Resource 2). In addition, the CRT decreased by −40.50 μm among Asian patients (*P* < 0.001) while VA remained stable, with +4.52 letters on average in this group (*P* < 0.001).

### Safety data

Of 3445 patients, 484 (14.0%) had at least one AE reported in their medical records. Eye disorders were the most common AEs reported (*n* = 293, 8.5%), followed by general disorders and administration site conditions (*n* = 53, 1.5%). Injury, poisoning and procedural complications were reported in 1.5% of patients (*n* = 52), infections and infestations in 1.5% (*n* = 50), and nervous system disorders in 1.0% (*n* = 36).

Eye disorders were more often reported in the self-paid group (*n* = 46, 21.9%) than in the other groups, notably conjunctival hemorrhage (*n* = 16, 7.6%), retinal detachment (*n* = 13, 6.2%), and vitreous hemorrhage (*n* = 8, 3.8%) (Table 5).

**Table 5:** Safety outcomes: eye disorders

Adverse events	Ranibizumab medical coverage			All combined (*n* = 3445)
	Fully-reimbursed (*n* = 568)	Partially-reimbursed (*n* = 2666)	Self-paid (*n* = 210)	
Eye disorders no. (%)	55 (9.7%)	192 (7.2%)	46 (21.9%)	293 (8.5%)
Cataract	3 (0.5%)	46 (1.7%)	5 (2.4%)	54 (1.6%)
Conjunctival hemorrhage	8 (1.4%)	19 (0.7%)	16 (7.6%)	43 (1.2%)
Retinal detachment	4 (0.7%)	7 (0.3%)	13 (6.2%)	24 (0.7%)
Dry eye	6 (1.1%)	14 (0.5%)	0 (0.0%)	20 (0.6%)
Detachment of retinal pigment epithelium	2 (0.4%)	8 (0.3%)	4 (1.9%)	14 (0.4%)
Vitreous hemorrhage	2 (0.4%)	3 (0.1%)	8 (3.8%)	13 (0.4%)
Retinal hemorrhage	5 (0.9%)	1 (0.0%)	6 (2.9%)	12 (0.3%)
Eye hemorrhage	2 (0.4%)	4 (0.2%)	4 (1.9%)	10 (0.3%)
Conjunctivitis	1 (0.2%)	5 (0.2%)	2 (1.0%)	8 (0.2%)
Trichiasis	1 (0.2%)	3 (0.1%)	4 (1.9%)	8 (0.2%)
Cystoid macular edema	0 (0.0%)	3 (0.1%)	2 (1.0%)	5 (0.1%)
Eye discharge	2 (0.4%)	1 (0.0%)	2 (1.0%)	5 (0.1%)
Macular scar	2 (0.4%)	1 (0.0%)	2 (1.0%)	5 (0.1%)
Polypoidal choroidal vasculopathy	0 (0.0%)	3 (0.1%)	2 (1.0%)	5 (0.1%)
Choroidal neovascularization	1 (0.2%)	0 (0.0%)	2 (1.0%)	3 (0.1%)
Retinal depigmentation	1 (0.2%)	0 (0.0%)	2 (1.0%)	3 (0.1%)
Retinal scar	0 (0.0%)	1 (0.0%)	2 (1.0%)	3 (0.1%)

### Concomitant nAMD therapy and switch to another anti-VEGF

Of 333 eyes (8.9% overall) with a concomitant nAMD therapy, 58.3% (*n* = 194) of eyes were treated with PDT, and 47.1% (*n* = 157) received either laser, steroid, surgery, or other treatment (Table 6).

**Table 6:** Concomitant nAMD therapy

Concomitant therapy	Ranibizumab medical coverage			All combined (*n* = 3725)
	Fully-reimbursed (*n* = 580)	Partially-reimbursed (*n* = 2945)	Self-paid (*n* = 200)	
Eyes treated, no. (%)	88 (15.2%)	164 (5.6%)	81 (40.5%)	333 (8.9%)
Therapy, no. (%)				
Verteporfin photodynamic therapy	61 (10.5%)	67 (2.3%)	66 (33.0%)	194 (5.2%)
Laser	12 (2.1%)	30 (1.0%)	5 (2.5%)	47 (1.3%)
Steroid	6 (1.0%)	29 (1.0%)	0	35 (0.9%)
Surgery	2 (0.3%)	21 (0.7%)	6 (3.0%)	29 (0.8%)
Other	10 (1.7%)	30 (1.0%)	6 (3.0%)	46 (1.2%)

*nAMD*, neovascular age-related macular degeneration

At the end of the observation period, 341 eyes (9.2% overall) were switched to another anti-VEGF treatment, of which 73.3% (*n* = 250) were switched to bevacizumab and 26.7% (*n* = 91) to aflibercept (Table 7). A balanced distribution of switches between bevacizumab and aflibercept was observed in the fully-reimbursed group.

**Table 7:** Switch to another anti-VEGF

Therapy switch	Ranibizumab medical coverage			
	Fully-reimbursed (*n* = 580)	Partially-reimbursed (*n* = 2945)	Self-paid (*n* = 200)	All combined (*n* = 3725)
Eyes switching, no. (%)	36 (6.2%)	294 (10.0%)	11 (5.5%)	341 (9.2%)
Reason for switch, no. (%)				
Cost	6 (1.0%)	125 (4.2%)	4 (2.0%)	135 (3.6%)
Unknown	8 (1.4%)	97 (3.3%)	3 (1.5%)	108 (2.9%)
Efficacy	18 (3.1%)	58 (2.0%)	1 (0.5%)	77 (2.1%)
Patient preference	2 (0.3%)	13 (0.4%)	6 (3.0%)	21 (0.6%)
Other	1 (0.2%)	11 (0.4%)	0	12 (0.3%)
Safety/tolerability	0	3 (0.1%)	0	3 (0.1%)
Clinical trial participation	1 (0.2%)	1 (0.0%)	0	2 (0.1%)
Drug accessibility (other than cost-related)	0	2 (0.1%)	0	2 (0.1%)
Therapy taken, no. (%)				
Bevacizumab	17 (2.9%)	224 (7.6%)	9 (4.5%)	250 (6.7%)
Aflibercept	19 (3.3%)	70 (2.4%)	2 (1.0%)	91 (2.4%)

*Anti-VEGF*, anti-vascular endothelial growth factor

Of patients who switched to another anti-VEGF (*n* = 341), the most frequently recorded reason for switching was cost (*n* = 135, 39.6%). The same reason was observed to be the most frequent in the partially-reimbursed group. In the self-paid group (*n* = 11 switching to another anti-VEGF), patient preference was reported as the reason for switch in 54.5% of eyes (*n* = 6), and cost in 36.4% (*n* = 4). In the fully-reimbursed group (*n* = 36 switching to anti-VEGF), efficacy was reported as the main reason for switch for half of the eyes (*n* = 18) (Table 7).

### Subgroup results

There were 1166 patients and 1253 eyes in subgroup A (patients with last ranibizumab injection 13–24 months after first ranibizumab injection, 36.0% of analysis population). Subgroup B was smaller, with 719 patients and 767 eyes (patients with last ranibizumab injection 25–36 months after first ranibizumab injection, 22.2% of analysis population). The remaining 1356 patients were treated for 1 year or less, but were observed for at least 1 year.

Most patients in subgroups A and B were partially-reimbursed for ranibizumab. In subgroup B specifically, 24.2% (*n* = 174) of patients were fully-reimbursed, and 2.9% (*n* = 21) were self-paid.

Prior to the ranibizumab initiation, 12.8% (*n* = 160) of patients received nAMD treatment in subgroup A, compared to 23.3% (*n* = 179) in subgroup B. Previous nAMD treatment (mainly another anti-VEGF or PDT) was most common in the self-paid group.

Compared to the overall eye-population, subgroup A received 1 additional injection per year on average, while subgroup B received 1.6 additional injections per year. Patients in the self-paid group had the lowest mean yearly injection frequency in both subgroups (approximately 4 injections per year) (Fig. 1). Patients in subgroup A were more frequently monitored (1.5 additional visits per year on average) than patients in subgroup B (Table 4).

The mean VA change from baseline was lower than 5 letters in subgroups A and B (−1.5 [± 21.96] and −2.5 [± 21.65] letters respectively). Similar mean VA changes (no change or loss lower than 5 letters) were observed across all reimbursement categories (Fig. 2). A CRT decrease was observed in both subgroups and within all reimbursement categories; mean CRT change was −36.3 (± 136.54) and −25.2 (± 124.44) μm in subgroups A and B respectively (Fig. 3). The highest CRT decrease was observed in the self-paid category for both subgroups (−80.3 [± 231.20] and −52.8 [± 117.25] μm in subgroups A and B respectively).

## Discussion

This study, based on medical records, evaluated ranibizumab treatment, clinical monitoring frequencies, and visual outcomes of patients treated for nAMD in real-world settings in the Asia–Pacific region, the Middle East, and North Africa. Patients were categorized by three ranibizumab reimbursement scenarios (fully-reimbursed, partially-reimbursed, or self-paid). The partially-reimbursed group was the most prevalent reimbursement category in this study, with 2521 patients, corresponding to 2954 eyes. Markedly fewer patients were fully-reimbursed or self-paid for ranibizumab.

During the study period, patients were monitored approximately 7 times per year on average, irrespective of reimbursement category and ranibizumab dosing regimen. This was aligned with the findings from the AURA study (a retrospective observational study of treatment-naïve ranibizumab nAMD patients conducted in Europe, Canada and Venezuela) where patients had 7.5 monitoring visits and 9.8 clinic visits on average over 2 years of observation [17]. At the time of the study period, PRN and T&E were most commonly used in routine clinical practice, as such, the study population was probably receiving a mix of the two regimens. Oubraham et al. studied ranibizumab treatment-naïve patients under these two regimens during a 1-year period in which the number of monitoring visits was similar across those regimens, similar to the UNCOVER study. However, compared to the UNCOVER study population, the PRN group received approximately 1 more ranibizumab injection per year, the T&E group received approximately 4 more injections per year, and patients were monitored more frequently [18]. In a recent meta-analysis of 20 observational studies on ranibizumab treatment for nAMD mostly conducted in single countries in Europe, patients were receiving 1 more injection on average at 1 year compared to the UNCOVER population that was observed during a longer period [19]. The self-paid patients were treated less frequently on average (1 injection every 5 months) than patients who received any reimbursement (1 injection every 3 months), suggesting that reimbursement scenario can impact the number of injections received. In the AURA study, where almost the entire study population had a health insurance (93%) and got reimbursed for ranibizumab injections (95%), patients had received 5.4 injections on average at 1 year [17]. These findings correlate with the higher number of injections observed in the fully-reimbursed category in UNCOVER population.

The ranibizumab treatment frequency observed in the subgroups of patients with a longer treatment course (i.e., patients having more than 1-year treatment) echoes the findings of a study by Rayess et al. in which treatment-naïve patients starting T&E, ranibizumab, or bevacizumab received on average 5.7 and 5.8 injections in the second and third years of treatment [20].

In the present study, VA was maintained on average across all reimbursement categories with a relatively low ranibizumab dose regimen (every trimester on average) compared to monthly administration. Similarly, the meta-analysis which encompassed 11 among 20 observational studies with treatment-naïve patients estimated a change in VA at 1 year of +1.95 letters, weighted on the number of eyes treated [19]. In the AURA study, vision was maintained at 2 years in 67% of patients who were treatment-naïve [17]. Recent clinical trials showed that vision gains were maintained after 2 years in treatment-naïve populations under controlled PRN regimens [16, 21, 22]. Nonetheless, the UNCOVER study population encompassed both treatment-naïve and treatment-experienced patients, where treatment-naïve patients might be more likely to gain vision than treatment-experienced patients, who were more inclined to be in the maintenance phase. In addition, the variation of VA change results was greater (almost 10 letters) in our study compared to those published in clinical trials. A study conducted in Germany showed initial VA improvement that later declined after 4 months of ranibizumab treatment and tended toward baseline or lower in a PRN-treated population (having tour or more injections during the 12-month maintenance phase) [23].

These results were similar to the findings of the UNCOVER study, showing a weak correlation between the VA change and the frequency of ranibizumab injections.

The prognostic effects of selected variables on VA were also studied in the AURA population [24]. Similarly to the findings of the multivariate model in UNCOVER, the number of ranibizumab injections suggested to have a positive effect on VA.

The results observed for the two subgroups, being treated at least 1 or 2 years during the observation period, were consistent with those observed in the overall population. Interestingly, despite receiving injections more frequently, they lost slightly more letters on average than the overall population and showed greater variability. This can be explained by the size of the subgroups representing one-third and one-fifth of the overall eye-population assessed for the subgroups being treated at least 1 year and at least 2 years respectively.

Self-paid patients, who were mostly Asian, had the highest baseline CRT and experienced the greatest CRT decrease from baseline. They presented with more eye comorbidities than those in the two other reimbursement categories, notably PCV. Additionally, self-paid patients who had less than 1-year observation and thus, were ineligible for the study, may have been less likely to receive routine monitoring. Therefore, their visual outcomes may be different than those observed in the study.

Strengths of this study include the identification of a large, multinational, heterogeneous cohort of patients treated with ranibizumab injections, allowing for a description of ranibizumab treatment frequency and clinical outcomes across different reimbursement scenarios. A limitation inherent to the design of this study is the limited availability and completeness of data in the patient medical records, notably information on AEs. Therefore, AE frequency could have been underestimated.

## Conclusion

This study was the first of its kind to assess the effect of treatment reimbursement on ranibizumab use and treatment outcomes in routine clinical practice in a large heterogeneous population of ranibizumab-treated patients from the Asia–Pacific region, the Middle East, and North Africa. Patients were monitored at the same frequency irrespective of ranibizumab reimbursement status; however, more frequent ranibizumab treatment was observed in the fully-reimbursed and partially-reimbursed groups compared to the self-paid group. This may indicate that reimbursement scenario can impact the frequency of ranibizumab injections. No gain in VA was observed in the study population; this could be related to the relatively low ranibizumab injection frequencies observed in our study compared to standard regimens. Nevertheless, patients in all reimbursement scenarios attained vision stability on average, indicating control of disease activity.

## Electronic supplementary material

Online Resource 1 (PDF 14 kb)

Online Resource 2 (PDF 90 kb)
