# Supplementary material for: An analysis of ranibizumab treatment and visual outcomes in real-world settings: the UNCOVER study
Source: Graefes Arch Clin Exp Ophthalmol. 2018 Mar 3;256(5):963–73. doi: 10.1007/s00417-017-3890-8 (PMC5911274; doi:10.1007/s00417-017-3890-8)
Supplement: Supplementary file 1 — (PDF 14 kb) [file 417_2017_3890_MOESM1_ESM.pdf]

Online Resource 1

Multivariate regression model: Analysis of mean change in Visual Acuity Scores (in letters)

**Model parameter**	**Evaluable eyes**	**Class level**	**Estimate**	**Standard error**	**p-value**
Intercept			18.03	3.352	0.103
Ranibizumab frequency/year	3058		0.66	0.146	< 0.001
Medical coverage	444	Fully-reimbursed	1.51	1.854	0.412
	2448	Partially-reimbursed	0.26	1.174	0.822
	166	Self-paid	0.00		
Predominant race	1393	Caucasian	2.92	1.040	0.005
	983	Asian	4.52	1.170	< 0.001
	682	Other	0.00		
Sex	1547	Male	-1.57	0.798	0.049
	1511	Female	0.00		
Age	3058		-0.25	0.042	< 0.001
Duration of observational period	3058		-2.59	0.633	< 0.001
