# Supplementary material for: An analysis of ranibizumab treatment and visual outcomes in real-world settings: the UNCOVER study
Source: Graefes Arch Clin Exp Ophthalmol. 2018 Mar 3;256(5):963–73. doi: 10.1007/s00417-017-3890-8 (PMC5911274; doi:10.1007/s00417-017-3890-8)
Supplement: Supplementary file 2 — (PDF 90 kb) [file 417_2017_3890_MOESM2_ESM.pdf]

Online Resource 2

Multivariate regression model: Analysis of mean change in Central Retinal Thickness (in µm)

| **Model parameter** | **Evaluable eyes** | **Class level** | **Estimate** | **Standard error** | **p-value** |
|---|---|---|---|---|---|
| Intercept | | | -46.05 | 25.646 | 0.072 |
| Ranibizumab frequency/year | 2146 | | -1.04 | 1.125 | 0.357 |
| Medical coverage | 346 | Fully-reimbursed | -40.41 | 14.228 | 0.004 |
| | 1687 | Partially-reimbursed | -22.70 | 8.428 | 0.007 |
| | 113 | Self-paid | 0.00 | | |
| Predominant race | 947 | Caucasian | -18.28 | 9.013 | 0.042 |
| | 830 | Asian | -40.50 | 9.129 | < 0.001 |
| | 369 | Other | 0.00 | | |
| Sex | 1096 | Male | 11.45 | 6.123 | 0.062 |
| | 1050 | Female | 0.00 | | |
| Age | 2146 | | 0.02 | 0.318 | 0.952 |
| Duration of observational period | 2146 | | 15.70 | 4.875 | 0.001 |
